# Characterization of a novel lytic bacteriophage from an industrial *Escherichia coli* fermentation process and elimination of virulence using a heterologous CRISPR–Cas9 system

**DOI:** 10.1007/s10295-018-2015-7

**Published:** 2018-02-07

**Authors:** Mathew C. Halter, James A. Zahn

**Affiliations:** DuPont Tate & Lyle Bio Products, 198 Blair Bend Drive, Loudon, TN 37774 USA

**Keywords:** CRISPR, Cas9, Bacteriophage, White biotechnology, Industrial fermentation, 1,3-Propanediol, PDO

## Abstract

Bacterial–bacteriophage interactions are a well-studied and ecologically-important aspect of microbiology. Many commercial fermentation processes are susceptible to bacteriophage infections due to the use of high-density, clonal cell populations. Lytic infections of bacterial cells in these fermentations are especially problematic due to their negative impacts on product quality, asset utilization, and fouling of downstream equipment. Here, we report the isolation and characterization of a novel lytic bacteriophage, referred to as bacteriophage *DTL* that is capable of rapid lytic infections of an *Escherichia coli* K12 strain used for commercial production of 1,3-propanediol (PDO). The bacteriophage genome was sequenced and annotated, which identified 67 potential open-reading frames (ORF). The tail fiber ORF, the largest in the genome, was most closely related to bacteriophage *RTP*, a T1-like bacteriophage reported from a commercial *E. coli* fermentation process in Germany. To eliminate virulence, both a fully functional *Streptococcus thermophilus* CRISPR3 plasmid and a customized *S. thermophilus* CRISPR3 plasmid with disabled spacer acquisition elements and seven spacers targeting the bacteriophage *DTL* genome were constructed. Both plasmids were separately integrated into a PDO production strain, which was subsequently infected with bacteriophage *DTL*. The native *S. thermophilus* CRISPR3 operon was shown to decrease phage susceptibility by approximately 96%, while the customized CRISPR3 operon provided complete resistance to bacteriophage *DTL*. The results indicate that the heterologous bacteriophage-resistance system described herein is useful in eliminating lytic infections of bacteriophage *DTL*, which was prevalent in environment surrounding the manufacturing facility.

**Electronic supplementary material:**

The online version of this article (10.1007/s10295-018-2015-7) contains supplementary material, which is available to authorized users.

## Introduction

As the most abundant biological entity on the planet, bacteriophages play an important ecological role, and have also been exploited for the development of many modern technologies, including gene transfer and treatment of bacterial infections [1, 9, 10, 12]. On the other hand, owing to their ability to cause rapid lytic infections of bacterial cultures in a matter of minutes, the presence of bacteriophage in a modern industrial fermentation facility can be a serious problem, resulting in reduced product quality, loss in production capacity or asset utilization, and financial losses to the business. Lytic events in industrial fermentation can lead to periods of facility shut down for cleaning and elimination of bacteriophage, or even longer term shut down periods for redesign and modification of aseptic barriers in the facility. Bacteriophages are bacterial viruses, and with the steady increase in the use of prokaryotic bio-catalysts over the course of the last several decades for protein, small molecule, and chemical production, a focus has been placed on maintaining a bacteriophage-free environment in the manufacturing facility. As bacteriophages are not considered “living organisms” in the classical sense, they are often less susceptible to common sterilization practices [19] than their living hosts, exacerbating the problem of maintaining a sterile laboratory and working environment. Development of resistant production strains via classical strain improvement approaches [11, 27], as well as CRISPR-based acquired resistance systems [16], has become a common means to avoid the negative impacts of bacteriophage infection.

Chemical or UV-based random mutagenesis followed by target-based screening can often prove successful in producing bacteriophage-resistant production strains, but this approach involves the selection and screening of tens of thousands of colonies differing from the parent strain through one or more single-nucleotide polymorphisms (SNPs) in host genes related to the bacteriophage life cycle and/or virulence. This approach has potential for loss of bacteriophage resistance through further mutation of unstable modifications in host genes, or the mutation of bacteriophage genes [11, 27]. Alternatively, the use of CRISPR-based acquired resistance has become an attractive means to bypass random, non-targeted changes associated with classical strain improvement approaches. The Clustered Regularly Interspaced Palindromic Repeats (CRISPR) system was first discovered in *Streptococcus thermophilus* [2, 5, 7, 13, 15, 20], and provides prokaryotic acquired immunity against bacteriophage infection. Several proteins expressed from the operon are involved in recognizing the introduction of bacteriophage DNA, physically extracting a small stretch (~ 30 bp) of DNA from the infecting phage genome (a spacer), and inserting the spacer into spacer/repeat array of the operon, where it is continuously transcribed into, processed by nucleases into single repeat/spacer units (crRNA), and used by the CRISPR-specific RNA-guided nuclease, Cas9, as a targeting motif to seek out future phage DNA homologous to the ~ 30 bp spacer for degradation [4, 5, 14, 18]. Using this system, prokaryotes possess an acquired immunity against future infections by this specific phage [5, 6, 14, 24].

We recently isolated a novel bacteriophage capable of lytic infections of *Escherichia coli* K12 in a PDO production process. The 45,814 base pair genome was sequenced and annotated (GenBank accession: MG050172), and shown to be most similar to a phage isolated from an *E. coli* fermentation facility in Germany, RTP phage [28]. Two CRISPR-based bacteriophage immunity plasmids were then constructed, with one utilizing the entire functional operon, and the other utilizing a customized version targeted to seven different open-reading frames present in the bacteriophage genome that were deemed to be important based on homology to previously characterized bacteriophage genes. The full *S. thermophilus* CRISPR3 operon improved resistance to this novel bacteriophage by up to 96% via new spacer acquisition in plaque assays, whereas the customized CRISPR plasmid carrying the seven spacers known to target this phage genome did not allow for the formation of a single plaque across all biological and technical replicates. The results indicate that the heterologous bacteriophage-resistance system described herein is useful in eliminating lytic infections of bacteriophage *DTL*, which was a prevalent bacteriophage found in the environment surrounding the PDO-manufacturing facility.

## Materials and methods

### Isolation, sequencing, annotation, and phylogenetic analysis of phage DNA

The *E. coli* production strain utilized in experiments was a derivative of K12 FM5 (ATCC 53911). Crude bacterial/bacteriophage lysate was taken from a fermenter. The lysate was filtered through a 0.22-µm MCE membrane sterile filter to remove bacterial debris. The filtrate was then treated with DNase for 4 h to degrade any *E. coli* DNA present due to cell lysis while leaving bacteriophage DNA protected by the protein capsid. After DNA digestion, the DNase was heat inactivated, and the filtrate was then further treated with Proteinase K to remove the bacteriophage capsid and release the DNA into solution for isolation. After a 2-h proteinase treatment, protein was precipitated by treatment with 3 M potassium acetate. The flocculent was pelleted by centrifugation, and the bacteriophage DNA present in the supernatant was removed and precipitated using a 1:1 volume of 96% isopropyl alcohol. The precipitated nucleic acids were pelleted by centrifugation, and the supernatant was removed by pipetting. The DNA pellet was allowed to air dry to remove excess isopropyl alcohol, and then resuspended in double-distilled water (ddH_2_O).

Isolated DNA was sent to the DuPont Pioneer^®^ DNA Sequencing Facility (Johnston, IA,USA) for 454 pyrosequencing. A fully aligned 45,814 base pair contig was provided, which was then annotated using the online resource RAST (Rapid Annotation using Subsystem Technologies) [3, 8, 22]. The annotation produced a list of 67 potential open-reading frames (ORFs). Each ORF was queried using NCBI BLAST, and a predicted function was assigned based on the most significant match. All maps in this manuscript were created using Geneious version 10.2 (http://www.geneious.com) [17]. The tail fiber protein ORF, being the largest ORF present in the genome (3426 bp), was aligned against the most similar tail fiber protein ORFs present in NCBI, as well as a phiEB49 outgroup using ClustalW [26]. This alignment was imported into MEGA 6.06 [25] and used for phylogeny reconstruction using the neighbor-joining statistical method with 1000 bootstrap tests and a p-distance model. A 70% bootstrap reliability was used to hide unsupported branches.

### Transmission electron microscopy

Microscopy was performed by the Advanced Microscopy and Imaging Center, at the Joint Institute for Advanced Materials (University of Tennessee, Knoxville). High titer bacteriophage particles were isolated by PEG precipitation, stained with potassium phosphotungstic acid (KPTA) or uranyl acetate (UAc), respectively, and imaged using a Zeiss Libra 200 HT FE MC transmission electron microscope.

### *Streptococcus thermophilus* CRISPR3 Vector construction and transformation

*Streptococcus thermophilus* (LMD-9) (ATCC: BAA-491) was purchased from ATCC (American-Type Culture Collection). Cultures were grown in M17 broth (Oxoid) supplemented with 0.5% lactose. Following overnight growth, after confirmation by gram staining, 1 mL of culture was pelleted by centrifugation, resuspended in 500 µL of ddH_2_O, and treated with lysozyme to lyse the gram positive cells. Protein present in the lysate was precipitated by treatment with 3 M potassium acetate and pelleted by centrifugation. The resulting supernatant was removed, and DNA present was precipitated using a 1:1 volume of 96% isopropyl alcohol. The precipitated nucleic acids were pelleted by centrifugation, and the supernatant was removed by pipetting. The DNA pellet was allowed to air dry to remove excess isopropyl alcohol, and then resuspended in ddH_2_O.

#### Native *S. thermophilus* CRISPR3 plasmid

The full CRISPR/Cas9 locus from *S. thermophilus* (LMD-9) (7373 bp) was cloned into pACYC184 (New England Biolabs) following the multi-step amplification and sub-cloning procedure exactly as described by Sapranauskas et al. [24], in Supplementary Figure 1.

**Fig. 1:**
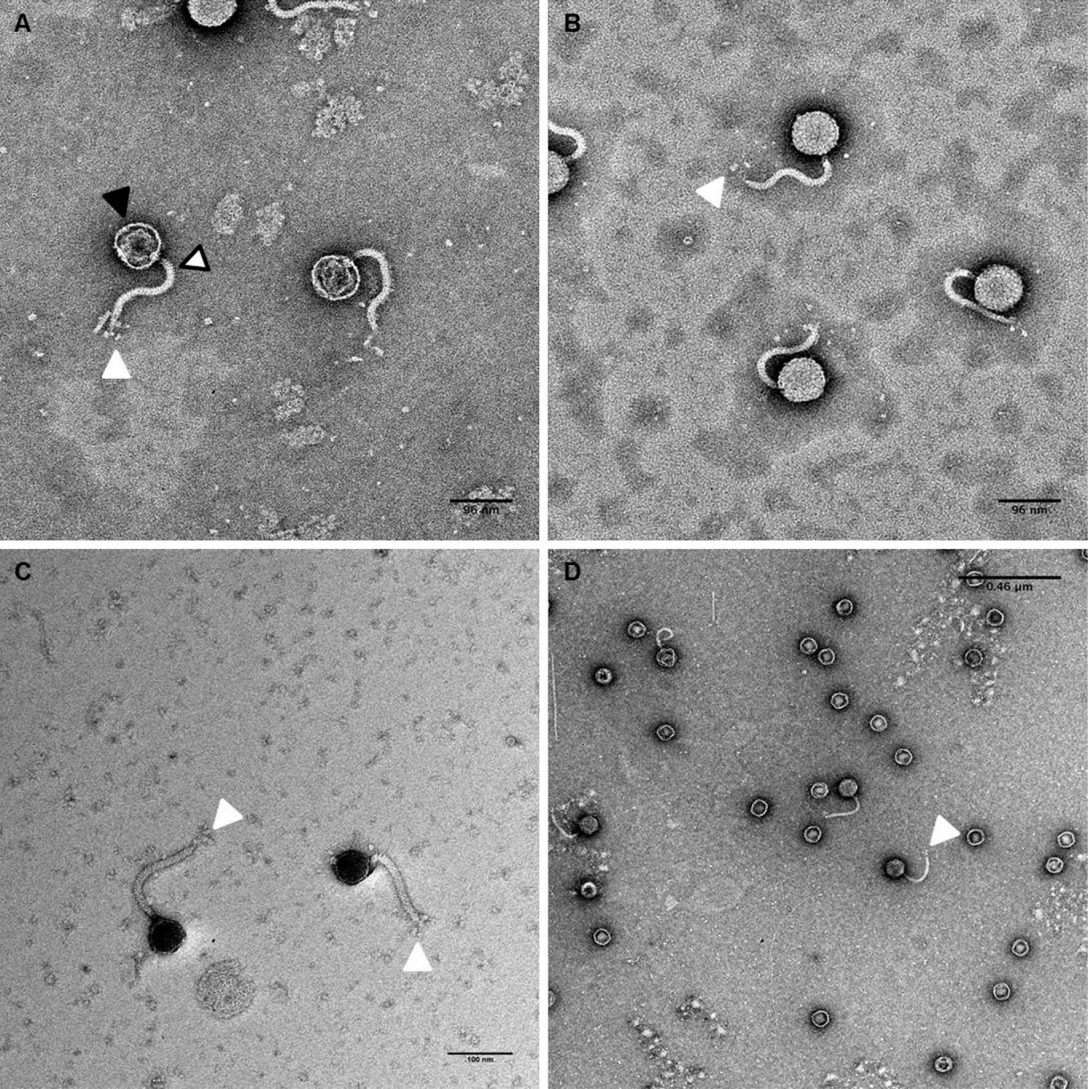
Transmission electron microscope micrographs of bacteriophage *DTL* isolated from lysed fermentation cultures. The solid black arrow identifies the capsid head, the white arrow/black border identifies the phage tail, and the white arrow identifies the rosette-style tail fibers. **a** 96-nm scale bar (KPTA stain), **b** 96-nm scale bar (KPTA stain), **c** 100-nm scale bar (UAc stain), **d** 0.46-µm scale bar (UAc stain)

#### Custom *S. thermophilus* CRISPR3 plasmid

The ability of the CRISPR3 operon to add new spacers was removed to prevent interference with secondary plasmids present in the *E. coli* production organism. Primers were designed to amplify the Cas9 ORF from upstream of the TracR RNA. The linker region then had the seven custom spacers/repeats added sequentially by extension PCR. The linker region fused to the new spacer/repeats was then fused to the end of the TracR/Cas9 region, again by extension/overlapping PCR. An in-depth description of primer sequences and the step-by-step process of creating the synthetic spacers/repeats can be found in the supplementary material associated with this manuscript. The resulting custom fusion gene contained upstream *Sal*I and downstream *Bam*HI cut sites. The fragment and pACYC184 were digested with *Sal*I and *Bam*HI, before being ligated together by an overnight treatment with DNA Ligase at 4 °C (See Supplementary Data for detailed procedure).

Both the native and the custom CRISPR3 plasmid were transformed into chemically competent Top10 Cells (Invitrogen), and 5-mL cultures were prepped for sequence confirmation of the plasmids prior to plaque assays. Plasmids confirmed by sequencing were transformed via electroporation *E. coli* K12 production strain. Positive colonies were selected for on chloramphenicol (pACYC184), and PCR screened using primers for the plasmid backbone and the CRISPR3 insert. Two colonies were selected for each plasmid assay to account for potential genetic differences associated with phage resistance.

### Plaque assays

Crude phage lysate was filter purified using a 0.22-µm MCE membrane sterile filter to remove bacterial debris. Serial dilutions were then performed to find a useful working concentration of plaque forming units (PFU) per milliliter of fermentation broth. The appropriate dilution from crude lysate, 10^−3^ PFU/mL produced an easily countable number of plaques when plated with production strain on LB media. Overnight cultures of production strain control, as well as two clonal CRISPR3 production strain lines were grown using chloramphenicol selection. In triplicate, 100 µL of each culture was inoculated with 1 µL of phage dilution and plated on LB. The resulting bacterial lawn clearly displayed plaque formation after overnight growth at 37 °C. All platings were done in triplicate.

## Results and discussion

Bacteriophage contamination in an industrial fermentation setting has the potential to cause reduced product quality, loss in production capacity or asset utilization, and financial losses. While preventing modes of contamination is pursued diligently, the total and complete prevention of bacteriophage entry into production fermentors is often a difficult task due to the challenges of maintaining the integrity of aseptic barriers in these axenic fermentation processes. Fermentation processes associated with White Biotechnology achieve a loss rate originating from biological contamination from between 0.1 and 5%; this loss can be attributed to the failure in systems designed to maintain the aseptic barrier, or the quality of inoculum fed into the fermentation process. Many bacteriophages demonstrate susceptibility to heat inactivation when compared to vegetative bacterial cells, and are often slightly less resistant to heat inactivation than Gram + spores, which can also be a common type of bacterial contamination in these processes [19]. In addition, the relative bioburden of bacteriophage contamination in a commercial fermentation environment can be significantly elevated by a single lytic phage event, where as many as 1 × 10^13^ PFU/mL can be produced by the fermentation process, and these particles can then be unintentionally disseminated through the plant environment through fermentor sampling, filter breakthrough, sparge gas atomization, and downstream handling of the broth. Finally, the small size of bacteriophages permits passage through filtration systems used for liquid service, and gas-phase service under conditions of high humidity or water/condensate entrainment. These factors underline the importance of a bacteriophage surveillance, a process hygiene program that specifically includes mitigation of bacteriophage transfer in the plant environment, and bacteriophage-resistance programs that drive down the failure rate associated with biological contamination in the production fermentor.

The ecological aspects of DTL bacteriophage are poorly characterized due to the lack of historical environmental samples from the fermentation facility during the period on initial infection. The fact that DTL phage is highly prevalent in the plant environment is thought to be mainly a result of a series of lytic events in production fermentors that significantly expanded localized levels of the bacteriophage. Each fermentor that is impacted by a lytic event can contain as much as 600,000 L of broth with plaque forming units approaching 1 × 1013 pfu/mL. Bacteriophage from these fermentors can be unintentionally disseminated through fermentor sampling, aerosolization, downstream processing, and disposal of contaminated broth. Although we have investigated many potential reservoirs including process air headers, human reservoirs, cooling tower basins, fermentation raw materials, and water supplies, the origin of this bacteriophage remains poorly understood.

The authors have identified a novel bacteriophage, bacteriophage *DTL* that was isolated from an industrial fermentation process, and was capable of rapid lytic infection of a strain of *E. coli* K12 used in the commercial manufacture of 1,3-propanediol. The tail fiber ORF, the largest in the genome, was most closely related to bacteriophage RTP, a T1-like bacteriophage reported from a commercial *E. coli* fermentation process in Germany [28]. These T1-like bacteriophages have similarities with the shape of the capsid head, which is attached to a long, flexible tail, and rosette-style fibers at the end of the tail (Fig. 1).

**Fig. 2:**
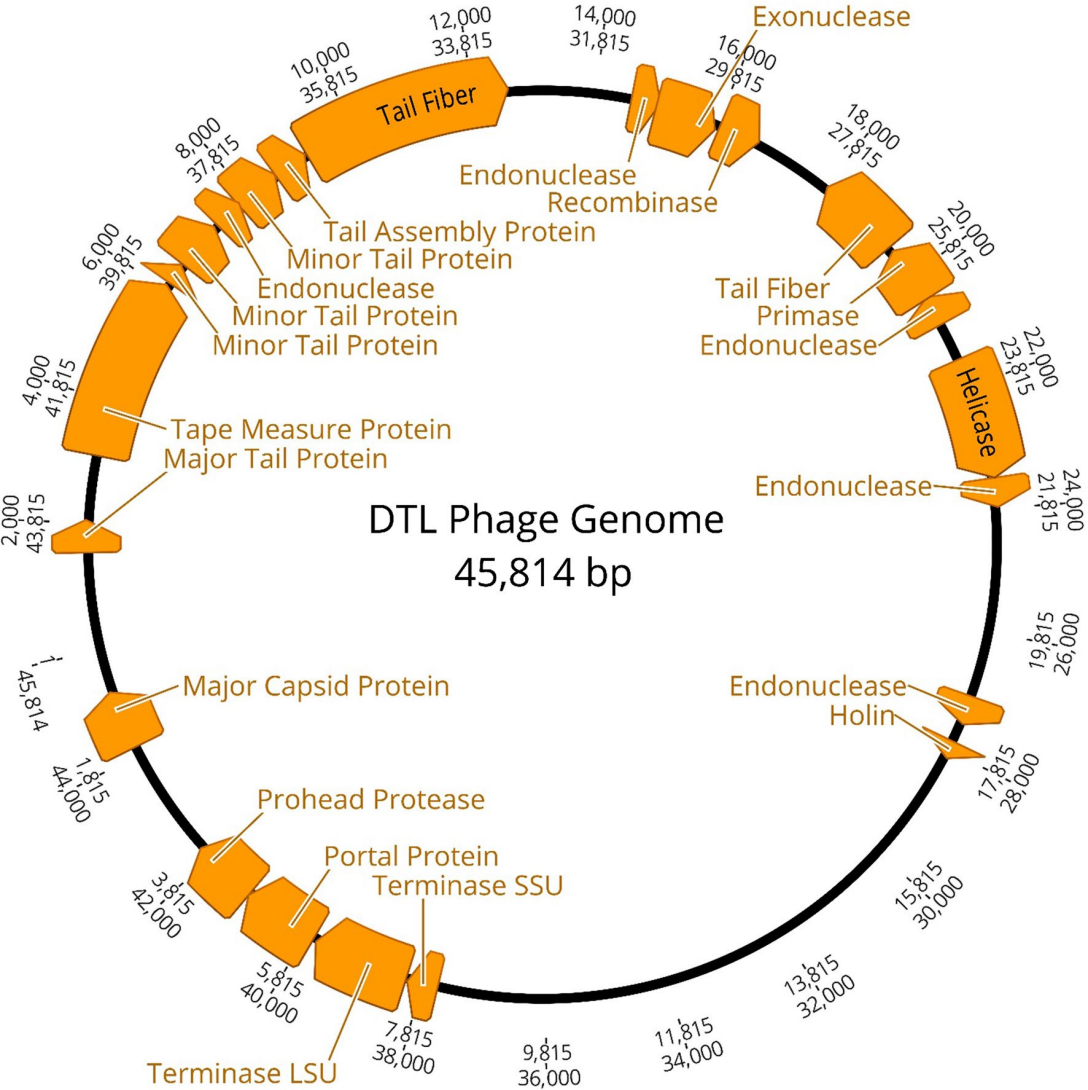
Bacteriophage *DTL* genome. Annotated open-reading frames with a predicted function identified

**Fig. 3:**
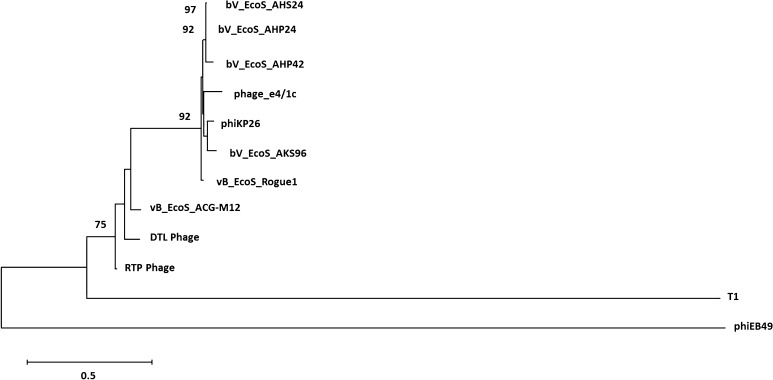
Neighbor joining trees generated from the multiple sequence alignments of the nucleotide sequences of the tail fiber protein genes of bacteriophage *DTL* (ORF 50), phiEB49 (outgroup), T1 phage, and several other highly similar bacteriophage tail fiber protein sequences found by NCBI BLAST. Bootstrap values appear at branch points

The genome of bacteriophage *DTL* was sequenced, revealing approximately 46,000 base pairs (Fig. 2) and 67 open-reading frames (Table 1). Of the 67 ORFs, 25 aligned closely to a known bacteriophage gene in NCBI, which was used to infer a function. It is not known whether all of these ORFs are directly or indirectly involved in the virulence and life cycle, or how many of them are expressed. The largest ORF present in the genome, that of the tail fiber protein (3426 bp), was queried with NCBI BLAST (National Center for Biotechnology Information, Basic Local Alignment Search Tool). Sequences for 11 phage tail fiber proteins that aligned closely to bacteriophage *DTL* tail fiber protein, as well as that of T1-phage, were extracted from NCBI and used to create a multiple sequence alignment (MSA) with ClustalW. The MSA was then used to create a phylogenetic tree in MEGA 6.06 (Fig. 3). The tree confirms closest relation to RTP phage, with fairly significant deviation from T1 phage. This deviation, especially in this gene, may explain the slight phenotypic differences referenced by Wietzorrek et al. [28], which can also be seen in Fig. 1 micrographs, in which the tail does taper off towards the end, where the tail fibers are attached. This virulent T1-like bacteriophage can be assigned into the previously proposed Tunavirinae subfamily and Rtplikevirus genus [21, 23].

**Table 1:** List of open-reading frames annotated within the bacteriophage DTL genome

ORF	ORF size (bp)	Strand	Predicted function	Significant match (organism) (protein sequence ID)	*E* value
1	249	+	Unknown	Hypothetical protein ACG-M12_0058 (Enterobacteria phage vB_EcoS_ACG-M12) (YP_006987877.1)	1.00E−50
2	252	+	Unknown	Hypothetical protein ACG-M12_0059 [Enterobacteria phage vB_EcoS_ACG-M12] (YP_006987878.1)	5.00E−47
3	1137	+	Unknown	Hypothetical protein rtp61 [Escherichia phage Rtp] (YP_399005.1)	0
4	474	+	Endonuclease	Putative HNH endonuclease [Escherichia phage Rtp] (YP_398984.1)	5.00E−30
5	177	+	Unknown	Hypothetical protein ACG-M12_0064 [Enterobacteria phage vB_EcoS_ACG-M12] (YP_006987883.1)	5.00E−34
6	294	+	Holin	Putative holin (Escherichia phage Rtp) (YP_399007.1)	7.00E−39
7	486	+	Endolysin	Putative endolysin (Enterobacteria phage vB_EcoS_ACG-M12) (YP_006987885.1)	9.00E−95
8	363	+	Unknown	Hypothetical protein rtp65 (Escherichia phage vB_Rtp) (YP_399009.1)	1.00E−74
9	336	−	Unknown	Hypothetical protein ACG-M12_0068 (Enterobacteria phage vB_EcoS_ACG-M12) (YP_006987887.1)	2.00E−66
10	1584	−	Unknown	Hypothetical protein rtp67 (Escherichia phage Rtp) (YP_399011.1)	0
11	354	−	Unknown	Hypothetical protein rtp69 (Escherichia phage Rtp) (YP_399013.1)	1.00E−65
12	168	−	Unknown	Hypothetical protein ACG-M12_0072 (Enterobacteria phage vB_EcoS_ACG-M12) (YP_006987891.1)	2.00E−22
13	522	−	Unknown	AP2 domain protein (Serratia ureilytica) (KKO5800.1)	3.00E−25
14	240	−	Unknown	Hypothetical protein rtp73 (Escherichia phage Rtp) (YP_399017.1)	6.00E−48
15	177	−	Unknown	Hypothetical protein kp_75 (Enterobacterio phage phiKP26) (AGH25217.1)	1.00E−24
16	480	+	Unknown	Hypothetical protein (Escherichia phage e4/1c) (YP_009036062.1)	2.00E−18
17	423	+	Unknown	Hypothetical protein AKS96_64 (Escherichia phage bV_EcoS_AKS96) (YP_009056119.1)	4.00E−44
18	252	+	Unknown	Hypothetical protein SP126_00225 (Salmonella phage FSL SP-126) (AGF87875.1)	3.00E−17
19	384	+	Unknown	Hypothetical protein SP126_00225 (Salmonella phage FSL SP-126) (AGF87875.1)	6.00E−23
20	231	+	Unknown	Hypothetical protein ACG-M12_0005 (Enterobacteria phage vB_EcoS_ACG-M12) (YP_006987823.1)	8.00E−26
21	150	+	Unknown	Hypothetical protein Shfl1p78 [Shigella phage Shfl1) (YP_004414891.1)	1.00E−09
22	291	+	Unknown	Hypothetical protein rtp11 (Escherichia phage Rtp) (YP_398955.1)	4.00E−31
23	120	+	Unknown	Hypothetical protein rtp12 (Escherichia phage Rtp) (YP_398956.1)	7.00E−14
24	474	+	Endonuclease	HNH endonuclease (Vibrio phage pYD38-A) (YP_008126236.1)	4.00E−34
25	240	+	Unknown	Hypothetical protein ACG-M12_0012 (Enterobacteria phage vB_EcoS_ACG-M12) (YP_006987830.1)	2.00E−48
26	261	+	Unknown	Hypothetical protein ACG-M12_0013 (Enterobacteria phage vB_EcoS_ACG-M12) (YP_006987831.1)	9.00E−34
27	507	+	Terminase SSU	Putative terminase small subunit (Escherichia phage Rtp) (YP_398963.1)	4.00E−110
28	1572	+	Terminase LSU	Putative terminase large subunit (Escherichia phage Rtp) (YP_398965.1)	0
29	1266	+	Portal protein	gp56 (Escherichia phage EB49) (YP_009018670.1)	0
30	1089	+	Prohead protease	gp55 (Escherichia phage EB49) (YP_009018669.1)	0
31	525	+	Unknown	gp54 (Escherichia phage EB49) (YP_009018668.1)	6.00E−92
32	450	+	Unknown	gp53 (Escherichia phage EB49) (YP_009018667.1)	2.00E−84
33	177	+	Unknown	gp53 (Escherichia phage EB49) (YP_009018667.1)	1.00E−22
34	942	+	Major capsid protein	gp52 (Escherichia phage EB49) (YP_009018666.1)	0
35	246	+	Unknown	gp50 (Escherichia phage EB49) (YP_009018664.1)	2.00E−47
36	402	+	Unknown	Halo29 (Escherichia phage RES-2009a) (ACZ74599.1)	1.00E−89
37	372	+	Unknown	Halo30 (Escherichia phage RES-2009a) (ACZ74600.1)	3.00E−75
38	438	+	Unknown	Hypothetical protein ACG-M12_0024 (Enterobacteria phage vB_EcoS_ACG-M12) (YP_006987842.1)	6.00E−96
39	402	+	Unknown	Hypothetical protein ACG-M12_0025 (Enterobacteria phage vB_EcoS_ACG-M12) (YP_006987843.1)	6.00E−87
40	393	+	Major tail protein	Putative major tail protein (Enterobacteria phage vB_EcoS_ACG-M12) (YP_006987844.1)	4.00E−85
41	249	+	Unknown	Hypothetical protein ACG-M12_0027 (Enterobacteria phage vB_EcoS_ACG-M12) (YP_006987845.1)	1.00E−17
42	315	+	Unknown	Hypothetical protein rtp35 (Escherichia phage Rtp) (YP_398979.1)	2.00E−61
43	312	+	Unknown	Hypothetical protein rtp36 (Escherichia phage Rtp) (YP_39890.1)	6.00E−68
44	2976	+	Tape-measure protein	Putative tail tape-measure protein (Enterobacteria phage vB_EcoS_ACG-M12) (YP_006987848.1)	0
45	351	+	Minor tail protein	Putative minor tail protein (Escherichia phage e4/1c) (YP_009036021.1)	7.00E−68
46	756	+	Minor tail protein	Putative minor tail protein (Enterobacteria phage vB_EcoS_ACG-M12) (YP_006987851.1)	3.00E−130
47	486	+	Endonuclease	gp40 (Escherichia phage EB49) (YP_009018654.1)	6.00E−43
48	759	+	Minor tail protein	Putative minor tail protein (Enterobacteria phage vB_EcoS_ACG-M12) (YP_006987353.1)	1.00E−179
49	573	+	Tail assembly	Putative tail assembly protein (Enterobacteria phage vB_EcoS_ACG-M12)	1.00E−133
50	3426	+	Tail fiber	Putative tail fiber protein (Escherichia phage Rtp) (YP_398987.1)	0
51	957	−	Unknown	go33 (Escherichia phage EB49) (YP_009018647.1)	5.00E−157
52	198	−	Unknown	Hypothetical protein (Escherichia phage e4/1c) (YP_009036027.1)	1.00E−31
53	249	+	Unknown	Hypothetical protein rtp46 (Escherichia phage Rtp)	2.00E−35
54	474	+	Endonuclease	Homing endonuclease (Enterobacteria phage CAjan) (YP_009018673.1)	5.00E−46
55	978	+	Exonuclease	Putative exodeoxyribonuclease VIII (Enterobacteria phage vB_EcoS_ACG-M12) (YP_006987862.1)	0
56	651	+	Recombinase	Putative recombination protein (Escherichia phage Rtp) (YP_398992.1)	2.00E−148
57	423	+	Unknown	Putative single-stranded DNA binding protein (Escherichia phage Rtp) (YP_398993.1)	6.00E−72
58	1377	−	Tail fiber	Putative tail fiber (Escherichia phage Rtp) (YP_398994.1)	2.00E−62
59	924	−	Primase	Putative DNA primase (Enterobacteria phage vB_EcoS_ACG-M12) (YP_006987867.1)	3.00E−162
60	480	−	Endonuclease	HNH endonuclease (Vibrio phage pYD38-A) (YP_008126236.1)	4.00E−40
61	474	−	Unknown	Putative transcriptional regulator (Escherichia phage Rtp) (YP_398996.1)	2.00E−109
62	1995	+	Helicase	Putative ATP-dependent helicase (Escherichia phage Rtp) (YP_398997.1)	0
63	474	+	Endonuclease	gp40 (Escherichia phage EB49) (YP_009018654.1)	1.00E−45
64	420	+	Unknown	Hypothetical protein rtp54 (Escherichia phage Rtp) (YP_398998.1)	7.00E−91
65	195	+	Unknown	Hypothetical protein JK_68 (Escherichia phage Jk06) (YP_277508.1)	4.00E−3C
66	366	+	Unknown	Hypothetical protein ACG-M12_0054 (Enterobacteria phage vB_EcoS_ACG-M12) (YP_006987873.1)	4.00E−29
67	126	+	Unknown	Hypothetical protein rtp58 (Escherichia phage Rtp) (YP_399002.1)	1.00E−13

Chemical or UV-based random mutagenesis followed by target-based screening can often prove successful in producing bacteriophage-resistant production strains, but this approach involves the selection and screening of tens of thousands of colonies differing from the parent strain through one or more single-nucleotide polymorphisms (SNPs) in host genes related to the bacteriophage life cycle and/or virulence. This approach has potential for loss of bacteriophage resistance through further mutation of unstable modifications in host genes, or the mutation of bacteriophage genes [11, 27]. Our internal programs have utilized parallel approaches utilizing this strategy, as well as more targeted approaches focused on modification of docking biomolecules of the cell surface that are utilized by bacteriophages, and the CRISPR/Cas9-resistance system, which is the focus of this paper. Our classical mutation and screening program has generated mutants that have demonstrated resistance to DTL bacteriophage, but these mutants demonstrate considerable (18–90%) losses in 1,3-propanediol titer and production rate versus the parental strain. Efforts to characterize the single-nucleotide polymorphisms occurring in these bacteriophage-resistant strains have not been fully successful in identifying the cause(s) for reduced production of 1,3-propanediol; however, we have observed deleterious mutations occurring in dextrose uptake systems and genes involved with carbon metabolism in approximately 78% of these bacteriophage-resistant mutants. The targeted approach using the CRISPR/Cas9 bacteriophage-resistance system has been shown to have a lower impact on 1,3-propanediol productivity (Fig. 5e) when compared to the strains derived from the mutation and screening program. The CRISPR/Cas9 constructs reported in this study exhibit a 9% reduction in 1,3-propanediol productivity versus the control (Fig. 5e). Two efforts are under way to improve equivalence for productivity; they include evaluation of promoters to reduce intracellular levels of Cas9 levels, and chromosomal integration of genes to further characterize the plasmid burden.

The primary focus of this study was the development of CRISPR/Cas9 resistance to bacteriophage *DTL* in the *E. coli* PDO production strain, with a goal of achieving complete resistance via CRISPR spacer customization, rather than reliance on spacer acquisition. The CRISPR system evolved in prokaryotes to provide resistance against bacteriophage infection [14]. The sequence-specific nuclease activity of the Cas9 protein has recently been exploited for its ability to induce single-nucleotide polymorphisms (SNP) in target organisms for genetic modification. Here, we have utilized the CRISPR operon for its originally evolved function.

**Fig. 4:**
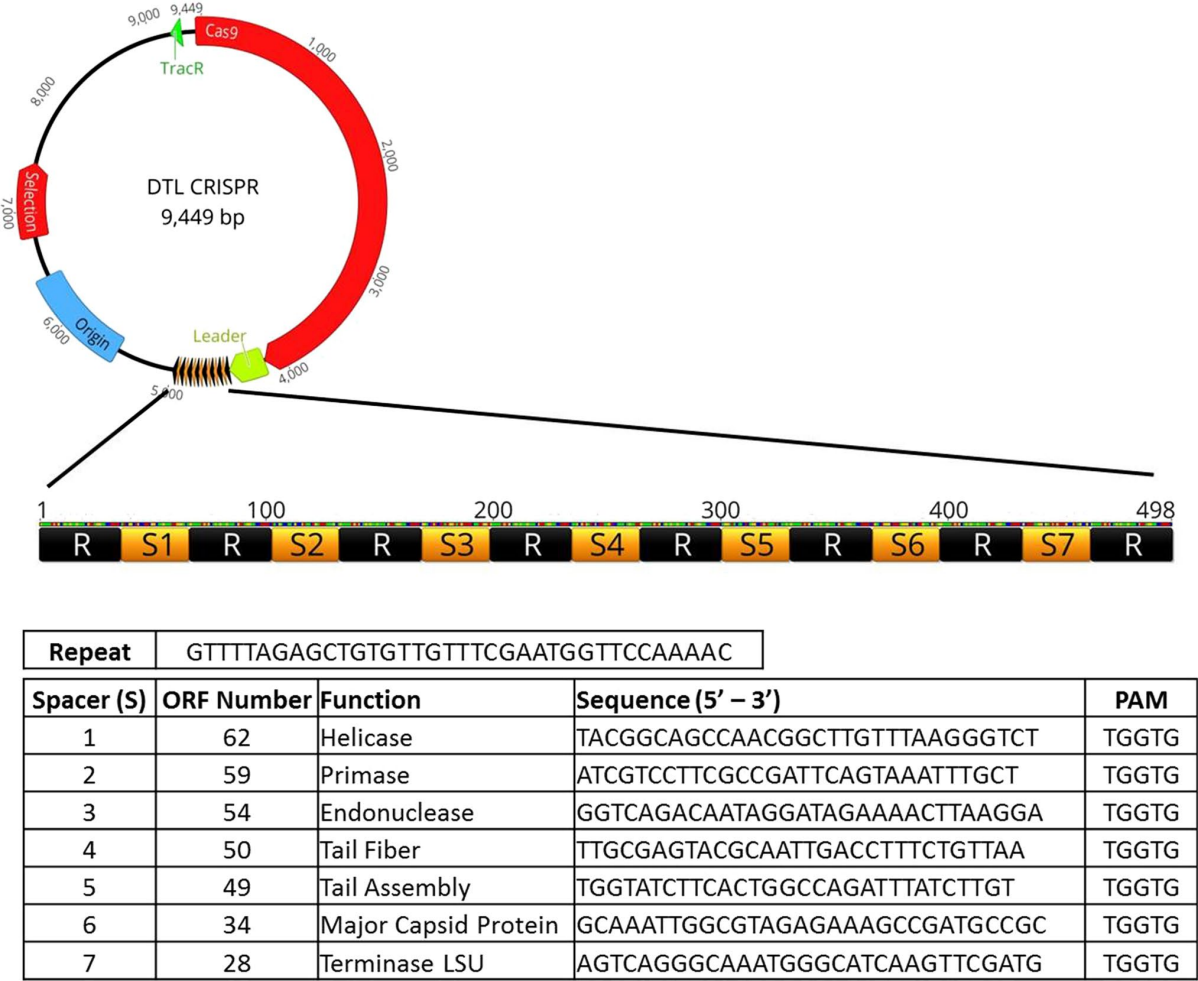
Schematic of the spacer/repeat sequence inserted into the custom CRISPR plasmid. Seven spacers were arbitrarily chosen based on (1) the presence of a PAM (proto-spacer adjacent motifsequence and (2) their assumed importance to the phage life cycle extrapolated from sequence analysis

**Fig. 5:**
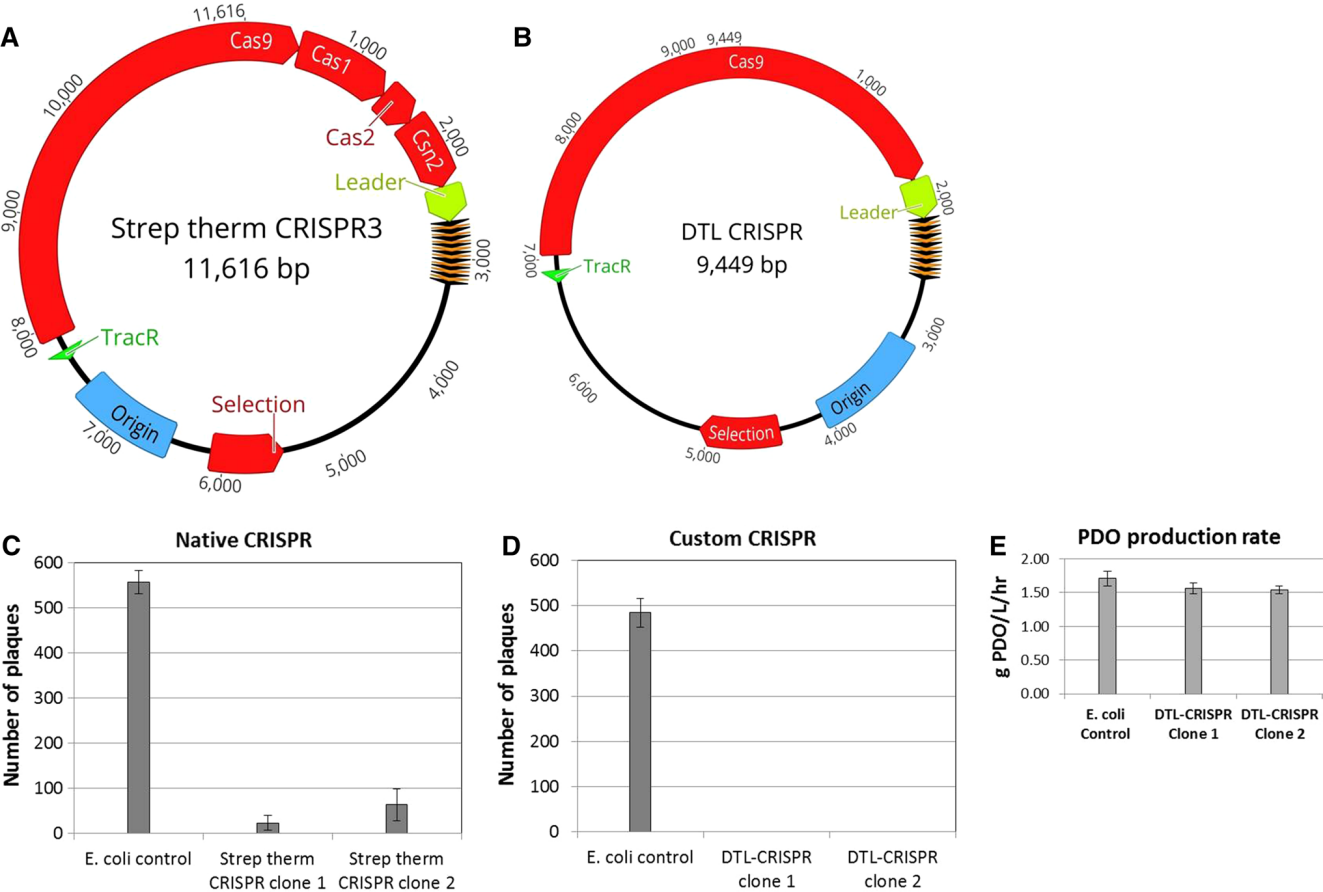
Plasmid schematics of the native *Streptococcus thermophilus* CRISPR3 plasmid (**a**) and the customized DTL-CRISPR plasmid (**b**). The native CRISPR3 still contains genes associated with new spacer acquisition, while the custom plasmid has had them removed, and tailored spacers inserted. Plaque assay data for both the native (**c**) and the custom (**d**) CRISPR plasmids. PDO production rates are shown for the clones expressing the DTL-CRISPR plasmid relative to the *E. coli* control (**e**)

The entire native *S. thermophilus* CRISPR3 sequence was cloned into a commonly available, low copy number plasmid, and transformed into *E. coli* K12 production strain. Two different pACYC184/CRISPR3 transformants were selected to ensure any resistance being gained was not due to genetic variation found among clonally selected bacterial strains. Liquid cultures were grown overnight and then incubated with bacteriophage *DTL* prior to plating. After a second night of growth, the plates indicated a 96 and 89% decrease in plaque number between the two pACYC184/CRISPR3 clones, respectively, relative to that of the production strain negative control (Fig. 5). This result is consistent with the previous reports, stating that *S. thermophilus* CRISPR3 is functional in *E. coli* [24]. This also exhibits a lag in full resistance associated with the need to acquire and implement spacers. To achieve full resistance, a new CRISPR3/Cas9 expression cassette was constructed, removing the ability to acquire new spacers, but replacing the native *S. thermophilus* spacers with seven spacers tailored to make cuts within what were deemed to be genes important for the phage life cycle (Fig. 4). These spacers were designed to bind next to a proto-spacer adjacent motif (5′-TGGTG-3′) located within the chosen gene of interest. This new cassette no longer requires the organism to spent valuable time integrating new spacers prior to gaining full-acquired resistance to a phage challenge. Again, transformation was performed and two production organism clones selected to account for genetic variation. These were assayed as described above, and no plaques were observed for either clone across triplicate plates (Fig. 5). This illustrates not only the strength of the acquired immunity against bacteriophage challenge post-spacer acquisition, but also the lag time associated with spacer acquisition during bacteriophage challenge. Evolutionarily, this fits a fairly standard natural selection model; those cells that can gain immunity quickest will survive. However, a significant loss of culture in a large-scale fermenter at any point can negatively impact the productivity and other financial metrics. New spacer acquisition also poses a risk to recombinant production strains, as the CRISPR3/Cas9 system has no way to differentiate between a recombinant plasmid and a circular, double-stranded DNA bacteriophage genome. Here, we have demonstrated that full resistance to this novel bacteriophage can be achieved in a PDO *E. coli* production strain while maintaining the ability to utilize production plasmids.

## Electronic supplementary material

Below is the link to the electronic supplementary material.

Supplementary material 1 (DOCX 36 kb)
