# Supplementary material for: Characterization of a novel lytic bacteriophage from an industrial Escherichia coli fermentation process and elimination of virulence using a heterologous CRISPR–Cas9 system
Source: J Ind Microbiol Biotechnol. 2018 Feb 7;45(3):153–63. doi: 10.1007/s10295-018-2015-7 (PMC5816109; doi:10.1007/s10295-018-2015-7)
Supplement: Supplementary file 1 — Supplementary material 1 (DOCX 36 kb) [file 10295_2018_2015_MOESM1_ESM.docx]

**Figures for “Characterization of a novel lytic bacteriophage from an industrial Escherichia coli fermentation process and elimination of virulence using a heterologous CRISPR‑Cas9 system”**

**Authors: Mathew C. Halter and James A. Zahn**

**Supplementary Data:**

**Construction of Custom DTL-CRISPR plasmid**

**Sequence of proposed custom linker/spacer cassette:**

TAAATTGGTAATAAGTATAGATAGTCTTGAGTTATTTCAAGACTATCTTTTAGTATTTAGTAGTTTCTGTATGAAGTTGAATGGGATAATCATTTTGTTAGAGAGTAGATTATAAGGATTTGATAGAGGAGGAATTAAGTTGCTTGACATATGATTATTAAGAAATAATCTAATATGGTGACAGTCACATCTTGTCTAAAACGTTGATATATAAGGATTTTTAAGGTATAATAAATATAAAAATGGAATTATTTTGAAGCTGAAGTCATGCTGAGATTAATAGTGCGATTACGAAATCTGGTAGAAAAGATATCCTACGAGGTTTTAGAGCTGTGTTGTTTCGAATGGTTCCAAAACTACGGCAGCCAACGGCTTGTTTAAGGGTCTGTTTTAGAGCTGTGTTGTTTCGAATGGTTCCAAAACATCGTCCTTCGCCGATTCAGTAAATTTGCTGTTTTAGAGCTGTGTTGTTTCGAATGGTTCCAAAACGGTCAGACAATAGGATAGAAAACTTAAGGAGTTTTAGAGCTGTGTTGTTTCGAATGGTTCCAAAACTTGCGAGTACGCAATTGACCTTTCTGTTAAGTTTTAGAGCTGTGTTGTTTCGAATGGTTCCAAAACTGGTATCTTCACTGGCCAGATTTATCTTGTGTTTTAGAGCTGTGTTGTTTCGAATGGTTCCAAAACAGTCAGGGCAAATGGGCATCAAGTTCGATGGTTTTAGAGCTGTGTTGTTTCGAATGGTTCCAAAACGCAAATTGGCGTAGAGAAAGCCGATGCCGCGTTTTAGAGCTGTGTTGTTTCGAATGGTTCCAAAACGGCCGCTCTGGTTGCTGTTGCTGCTGAAGCTTTTGTTATCACAATTTTCGGTTGACATCTCTTAGAACTCATCTTATCATAAAGGAGTCTAGTATTGAAATGTGAGAAGGGACATGTTATACGAATATCCAGC

Construction:

**Step 1.** Amplification of linker without spacers using primers:

F- TAAATTGGTAATAAGTATAGATAG

R- CTCGTAGGATATCTTTTCTAC

Resulting Sequence:

TAAATTGGTAATAAGTATAGATAGTCTTGAGTTATTTCAAGACTATCTTTTAGTATTTAGTAGTTTCTGTATGAAGTTGAATGGGATAATCATTTTGTTAGAGAGTAGATTATAAGGATTTGATAGAGGAGGAATTAAGTTGCTTGACATATGATTATTAAGAAATAATCTAATATGGTGACAGTCACATCTTGTCTAAAACGTTGATATATAAGGATTTTTAAGGTATAATAAATATAAAAATGGAATTATTTTGAAGCTGAAGTCATGCTGAGATTAATAGTGCGATTACGAAATCTGGTAGAAAAGATATCCTACGAG

**Step 2.** Addition of first spacer by extension PCR

F: TAAATTGGTAATAAGTATAGATAG

R: GTTTTGGAACCATTCGAAACAACACAGCTCTAAAACCTCGTAGGATATCTTTTCTAC

Underlined portion anneals.

Resulting sequence:

TAAATTGGTAATAAGTATAGATAGTCTTGAGTTATTTCAAGACTATCTTTTAGTATTTAGTAGTTTCTGTATGAAGTTGAATGGGATAATCATTTTGTTAGAGAGTAGATTATAAGGATTTGATAGAGGAGGAATTAAGTTGCTTGACATATGATTATTAAGAAATAATCTAATATGGTGACAGTCACATCTTGTCTAAAACGTTGATATATAAGGATTTTTAAGGTATAATAAATATAAAAATGGAATTATTTTGAAGCTGAAGTCATGCTGAGATTAATAGTGCGATTACGAAATCTGGTAGAAAAGATATCCTACGAGGTTTTAGAGCTGTGTTGTTTCGAATGGTTCCAAAAC

**Step 3.** Addition of second spacer by extension PCR

F: TAAATTGGTAATAAGTATAGATAG

R: AGC AAA TTT ACT GAA TCG GCG AAG GAC GAT GTT TTG GAA CCA TTC GAA ACA ACA CAG CTC TAA AAC AGA CCC TTA AAC AAG CCG TTG GCT GCC GTA GTT TTG GAA CCA TTC GAA

Underlined portion anneals

Resulting sequence:

TAAATTGGTAATAAGTATAGATAGTCTTGAGTTATTTCAAGACTATCTTTTAGTATTTAGTAGTTTCTGTATGAAGTTGAATGGGATAATCATTTTGTTAGAGAGTAGATTATAAGGATTTGATAGAGGAGGAATTAAGTTGCTTGACATATGATTATTAAGAAATAATCTAATATGGTGACAGTCACATCTTGTCTAAAACGTTGATATATAAGGATTTTTAAGGTATAATAAATATAAAAATGGAATTATTTTGAAGCTGAAGTCATGCTGAGATTAATAGTGCGATTACGAAATCTGGTAGAAAAGATATCCTACGAGGTTTTAGAGCTGTGTTGTTTCGAATGGTTCCAAAACTACGGCAGCCAACGGCTTGTTTAAGGGTCTGTTTTAGAGCTGTGTTGTTTCGAATGGTTCCAAAACATCGTCCTTCGCCGATTCAGTAAATTTGCT

**Step 4.** Addition of third spacer by extension PCR

F: TAAATTGGTAATAAGTATAGATAG

R: TCCTTAAGTTTTCTATCCTATTGTCTGACCGTTTTGGAACCATTCGAAACAACACAGCTCTAAA ACAGCAAATTTACTGAATCGGC

Resulting sequence:

TAAATTGGTAATAAGTATAGATAGTCTTGAGTTATTTCAAGACTATCTTTTAGTATTTAGTAGTTTCTGTATGAAGTTGAATGGGATAATCATTTTGTTAGAGAGTAGATTATAAGGATTTGATAGAGGAGGAATTAAGTTGCTTGACATATGATTATTAAGAAATAATCTAATATGGTGACAGTCACATCTTGTCTAAAACGTTGATATATAAGGATTTTTAAGGTATAATAAATATAAAAATGGAATTATTTTGAAGCTGAAGTCATGCTGAGATTAATAGTGCGATTACGAAATCTGGTAGAAAAGATATCCTACGAGGTTTTAGAGCTGTGTTGTTTCGAATGGTTCCAAAACTACGGCAGCCAACGGCTTGTTTAAGGGTCTGTTTTAGAGCTGTGTTGTTTCGAATGGTTCCAAAACATCGTCCTTCGCCGATTCAGTAAATTTGCTGTTTTAGAGCTGTGTTGTTTCGAATGGTTCCAAAACGGTCAGACAATAGGATAGAAAACTTAAGGA

**Step 5.** Addition of fourth spacer by extension PCR

F: TAAATTGGTAATAAGTATAGATAG

R: TTAACAGAAAGGTCAATTGCGTACTCGCAAGTTTTGGAACCATTCGAAACAACACAGCTCTAAAACTCCTTAAGTTTTCTATCCTATTG

Resulting sequence:

TAAATTGGTAATAAGTATAGATAGTCTTGAGTTATTTCAAGACTATCTTTTAGTATTTAGTAGTTTCTGTATGAAGTTGAATGGGATAATCATTTTGTTAGAGAGTAGATTATAAGGATTTGATAGAGGAGGAATTAAGTTGCTTGACATATGATTATTAAGAAATAATCTAATATGGTGACAGTCACATCTTGTCTAAAACGTTGATATATAAGGATTTTTAAGGTATAATAAATATAAAAATGGAATTATTTTGAAGCTGAAGTCATGCTGAGATTAATAGTGCGATTACGAAATCTGGTAGAAAAGATATCCTACGAGGTTTTAGAGCTGTGTTGTTTCGAATGGTTCCAAAACTACGGCAGCCAACGGCTTGTTTAAGGGTCTGTTTTAGAGCTGTGTTGTTTCGAATGGTTCCAAAACATCGTCCTTCGCCGATTCAGTAAATTTGCTGTTTTAGAGCTGTGTTGTTTCGAATGGTTCCAAAACGGTCAGACAATAGGATAGAAAACTTAAGGAGTTTTAGAGCTGTGTTGTTTCGAATGGTTCCAAAACTTGCGAGTACGCAATTGACCTTTCTGTTAA

**Step 6.** Addition of fifth spacer by extension PCR

F: TAAATTGGTAATAAGTATAGATAG

R: ACAAGATAAATCTGGCCAGTGAAGATACCAGTTTTGGAACCATTCGAAACAACACAGCTCTAA AACTTAACAGAAAGGTCAATTGCG

Resulting sequence:

TAAATTGGTAATAAGTATAGATAGTCTTGAGTTATTTCAAGACTATCTTTTAGTATTTAGTAGTTTCTGTATGAAGTTGAATGGGATAATCATTTTGTTAGAGAGTAGATTATAAGGATTTGATAGAGGAGGAATTAAGTTGCTTGACATATGATTATTAAGAAATAATCTAATATGGTGACAGTCACATCTTGTCTAAAACGTTGATATATAAGGATTTTTAAGGTATAATAAATATAAAAATGGAATTATTTTGAAGCTGAAGTCATGCTGAGATTAATAGTGCGATTACGAAATCTGGTAGAAAAGATATCCTACGAGGTTTTAGAGCTGTGTTGTTTCGAATGGTTCCAAAACTACGGCAGCCAACGGCTTGTTTAAGGGTCTGTTTTAGAGCTGTGTTGTTTCGAATGGTTCCAAAACATCGTCCTTCGCCGATTCAGTAAATTTGCTGTTTTAGAGCTGTGTTGTTTCGAATGGTTCCAAAACGGTCAGACAATAGGATAGAAAACTTAAGGAGTTTTAGAGCTGTGTTGTTTCGAATGGTTCCAAAACTTGCGAGTACGCAATTGACCTTTCTGTTAAGTTTTAGAGCTGTGTTGTTTCGAATGGTTCCAAAACTGGTATCTTCACTGGCCAGATTTATCTTGT

**Step 7.** Addition of sixth spacer by extension PCR

F: TAAATTGGTAATAAGTATAGATAG

R: CATCGAACTTGATGCCCATTTGCCCTGACTGTTTTGGAACCATTCGAAACAACACAGCTCTAA AACACAAGATAAATCTGGCCAG

Resulting sequence:

TAAATTGGTAATAAGTATAGATAGTCTTGAGTTATTTCAAGACTATCTTTTAGTATTTAGTAGTTTCTGTATGAAGTTGAATGGGATAATCATTTTGTTAGAGAGTAGATTATAAGGATTTGATAGAGGAGGAATTAAGTTGCTTGACATATGATTATTAAGAAATAATCTAATATGGTGACAGTCACATCTTGTCTAAAACGTTGATATATAAGGATTTTTAAGGTATAATAAATATAAAAATGGAATTATTTTGAAGCTGAAGTCATGCTGAGATTAATAGTGCGATTACGAAATCTGGTAGAAAAGATATCCTACGAGGTTTTAGAGCTGTGTTGTTTCGAATGGTTCCAAAACTACGGCAGCCAACGGCTTGTTTAAGGGTCTGTTTTAGAGCTGTGTTGTTTCGAATGGTTCCAAAACATCGTCCTTCGCCGATTCAGTAAATTTGCTGTTTTAGAGCTGTGTTGTTTCGAATGGTTCCAAAACGGTCAGACAATAGGATAGAAAACTTAAGGAGTTTTAGAGCTGTGTTGTTTCGAATGGTTCCAAAACTTGCGAGTACGCAATTGACCTTTCTGTTAAGTTTTAGAGCTGTGTTGTTTCGAATGGTTCCAAAACTGGTATCTTCACTGGCCAGATTTATCTTGTGTTTTAGAGCTGTGTTGTTTCGAATGGTTCCAAAACAGTCAGGGCAAATGGGCATCAAGTTCGATG

**Step 8.** Addition of seventh spacer by extension PCR

F: TAAATTGGTAATAAGTATAGATAG

R: GCGGCATCGGCTTTCTCTACGCCAATTTGCGTTTTGGAACCATTCGAAACAA CACAGCTCTAAAACCATCGAACTTGATGCCC

Resulting sequence:

TAAATTGGTAATAAGTATAGATAGTCTTGAGTTATTTCAAGACTATCTTTTAGTATTTAGTAGTTTCTGTATGAAGTTGAATGGGATAATCATTTTGTTAGAGAGTAGATTATAAGGATTTGATAGAGGAGGAATTAAGTTGCTTGACATATGATTATTAAGAAATAATCTAATATGGTGACAGTCACATCTTGTCTAAAACGTTGATATATAAGGATTTTTAAGGTATAATAAATATAAAAATGGAATTATTTTGAAGCTGAAGTCATGCTGAGATTAATAGTGCGATTACGAAATCTGGTAGAAAAGATATCCTACGAGGTTTTAGAGCTGTGTTGTTTCGAATGGTTCCAAAACTACGGCAGCCAACGGCTTGTTTAAGGGTCTGTTTTAGAGCTGTGTTGTTTCGAATGGTTCCAAAACATCGTCCTTCGCCGATTCAGTAAATTTGCTGTTTTAGAGCTGTGTTGTTTCGAATGGTTCCAAAACGGTCAGACAATAGGATAGAAAACTTAAGGAGTTTTAGAGCTGTGTTGTTTCGAATGGTTCCAAAACTTGCGAGTACGCAATTGACCTTTCTGTTAAGTTTTAGAGCTGTGTTGTTTCGAATGGTTCCAAAACTGGTATCTTCACTGGCCAGATTTATCTTGTGTTTTAGAGCTGTGTTGTTTCGAATGGTTCCAAAACAGTCAGGGCAAATGGGCATCAAGTTCGATGGTTTTAGAGCTGTGTTGTTTCGAATGGTTCCAAAACGCAAATTGGCGTAGAGAAAGCCGATGCCGC

**Step 9.** Addition of eighth spacer and terminator by extension PCR

F: TAAATTGGTAATAAGTATAGATAG

R: GCTGGATATTCGTATAACATGTCCCTTCTCACATTTCAATACTAGACTCCTTTAT GATAAGATGAGTTCTAAGAGATGTCAACCGAAAATTGTGATAACAAAAGCTTCAGCAGCAACAGCAACCAGAGCGGCCGTTTTGGAACCATTCGAAACAACACAGCTCTAAAACGCGGCATCGGCTTTCTC

Resulting sequence:

TAAATTGGTAATAAGTATAGATAGTCTTGAGTTATTTCAAGACTATCTTTTAGTATTTAGTAGTTTCTGTATGAAGTTGAATGGGATAATCATTTTGTTAGAGAGTAGATTATAAGGATTTGATAGAGGAGGAATTAAGTTGCTTGACATATGATTATTAAGAAATAATCTAATATGGTGACAGTCACATCTTGTCTAAAACGTTGATATATAAGGATTTTTAAGGTATAATAAATATAAAAATGGAATTATTTTGAAGCTGAAGTCATGCTGAGATTAATAGTGCGATTACGAAATCTGGTAGAAAAGATATCCTACGAGGTTTTAGAGCTGTGTTGTTTCGAATGGTTCCAAAACTACGGCAGCCAACGGCTTGTTTAAGGGTCTGTTTTAGAGCTGTGTTGTTTCGAATGGTTCCAAAACATCGTCCTTCGCCGATTCAGTAAATTTGCTGTTTTAGAGCTGTGTTGTTTCGAATGGTTCCAAAACGGTCAGACAATAGGATAGAAAACTTAAGGAGTTTTAGAGCTGTGTTGTTTCGAATGGTTCCAAAACTTGCGAGTACGCAATTGACCTTTCTGTTAAGTTTTAGAGCTGTGTTGTTTCGAATGGTTCCAAAACTGGTATCTTCACTGGCCAGATTTATCTTGTGTTTTAGAGCTGTGTTGTTTCGAATGGTTCCAAAACAGTCAGGGCAAATGGGCATCAAGTTCGATGGTTTTAGAGCTGTGTTGTTTCGAATGGTTCCAAAACGCAAATTGGCGTAGAGAAAGCCGATGCCGCGTTTTAGAGCTGTGTTGTTTCGAATGGTTCCAAAACGGCCGCTCTGGTTGCTGTTGCTGCTGAAGCTTTTGTTATCACAATTTTCGGTTGACATCTCTTAGAACTCATCTTATCATAAAGGAGTCTAGTATTGAAATGTGAGAAGGGACATGTTATACGAATATCCAGC

**Step 10.** Combination of Linkers and Cas9 by overhang PCR

Cas9 Forward: GTCATTGAGATCTTGGATGAGG

Cas9 Reverse: GACTATCTATACTTATTACCAATTTATTAACCCTCTCCTAGTTTGG

Cas9 3’ end:

AGACCTTGCCAAACTAGGAGAGGGTTAATAAATTGGTAATAAGTATAGATAGTC

Underlined anneals to the 5’ end of the linker sequence.


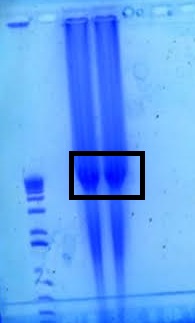


Overlap PCR was performed 20 cycles at Tm = 50.

The resulting band was gel purified, and confirmation PCR was performed using the Cas9 Forward primer above, as well as the RTP-linker reverse primer.

Cas9 Forward: GTCATTGAGATCTTGGATGAGG

RTP Linker R: GCTGGATATTCGTATAAC

The gel pictured above is of the confirmation PCR, 30 cycles, Tm = 57. This band was gel purified and ligated in pACYC184 cut by EcoRV

>Full sequence

ATGCGTCGACGTCATTGAGATCTTGGATGAGGTGGGAAAATAGTAGAGATAAAAGAGTCCTTTGGATGATTCCAAGGGACTCTTTGTGTATAAAAAAAACACCGAATCGGTGCCACCTTTTCAAGTTGAGTACGGACTAAGCCTTATTTTAACTCGCTGTGTTGTTTCGAATGGTTTCAAACCACAATTATTATTATATAGTTTTATAACTAATATGTCAATTTACTATAGGGTAAAGTATAAAAAATTTTATTCTTGATTACAAGATTTTAACGTCATACTATTTAATCAGTAATTCTATTAGATGTTGAAATATTGTTTAATAAGCGTATAATAATTTCTATAAATTTAGATTTTTCAAATAAGGAGAAATGTATGACTAAGCCATACTCAATTGGACTTGATATTGGAACGAATAGTGTTGGATGGGCTGTAACAACTGATAATTACAAGGTTCCGTCTAAAAAAATGAAAGTCTTAGGAAATACGAGTAAAAAGTATATCAAAAAGAACCTGTTAGGTGTATTACTCTTTGACTCTGGAATCACAGCAGAAGGAAGAAGATTGAAGCGTACTGCAAGAAGACGTTATACTAGACGCCGTAATCGTATCCTTTATTTGCAGGAAATTTTTAGCACAGAGATGGCTACATTAGATGATGCTTTCTTTCAAAGACTTGACGATTCGTTTTTAGTTCCTGATGATAAACGTGATAGTAAGTATCCGATATTTGGAAACTTAGTAGAAGAAAAAGCCTATCATGATGAATTTCCAACTATCTATCATTTAAGGAAATATTTAGCAGATAGTACTAAAAAAGCAGATTTGCGTCTAGTTTATCTTGCATTGGCTCATATGATTAAATATAGAGGTCACTTCTTAATTGAAGGAGAGTTTAATTCAAAAAATAATGATATTCAGAAGAATTTTCAAGACTTTTTGGACACTTATAATGCTATTTTTGAATCGGATTTATCACTTGAGAATAGTAAACAACTTGAGGAAATTGTTAAAGATAAGATTAGTAAATTAGAAAAGAAAGATCGTATTTTAAAACTCTTCCCTGGGGAGAAGAATTCGGGGATTTTTTCAGAGTTTCTAAAGTTGATTGTAGGAAATCAAGCTGATTTTAGGAAATGTTTTAATTTAGACGAAAAAGCCTCCTTACATTTTTCCAAAGAAAGCTATGATGAAGATTTAGAGACTTTGTTAGGTTATATTGGAGATGATTACAGTGATGTCTTTCTCAAAGCAAAGAAACTTTATGATGCTATTCTTTTATCGGGTTTTCTGACTGTAACTGATAATGAGACAGAAGCACCTCTCTCTTCTGCTATGATAAAGCGATATAATGAACACAAAGAAGATTTAGCGTTACTAAAGGAATATATAAGAAATATTTCACTAAAAACGTATAATGAAGTATTTAAAGATGACACCAAAAATGGTTATGCTGGTTATATTGATGGAAAAACAAATCAGGAAGATTTCTACGTATATCTAAAAAAACTATTGGCTGAATTTGAAGGTGCGGATTATTTTCTTGAAAAAATTGATCGAGAAGATTTTTTGAGAAAGCAACGTACATTTGACAATGGTTCGATACCATATCAGATTCATCTTCAAGAAATGAGAGCAATTCTTGATAAGCAAGCTAAATTTTATCCTTTCTTGGCTAAAAATAAAGAAAGAATCGAGAAGATTTTAACCTTCCGAATTCCTTATTATGTAGGTCCACTTGCGAGAGGGAATAGTGATTTTGCCTGGTCAATAAGAAAACGAAATGAAAAAATTACACCTTGGAATTTTGAGGACGTTATTGACAAAGAATCTTCGGCAGAGGCCTTCATTAATCGAATGACTAGTTTTGATTTGTATTTGCCAGAAGAGAAGGTACTTCCAAAGCATAGTCTCTTATACGAAACTTTTAATGTATATAATGAATTAACAAAAGTTAGATTTATTGCCGAAAGTATGAGAGATTATCAATTTTTAGATAGTAAGCAGAAGAAAGATATTGTTAGACTTTATTTTAAAGATAAAAGGAAAGTTACTGATAAGGATATTATTGAATATTTACATGCAATTTATGGGTATGATGGAATTGAATTAAAAGGCATAGAGAAACAGTTTAATTCTAGTTTATCTACTTATCACGATCTTTTAAATATTATTAATGATAAAGAGTTTTTGGATGATAGTTCAAATGAAGCGATTATCGAAGAAATTATCCATACTTTGACAATTTTTGAAGATAGAGAGATGATAAAACAACGTCTTTCAAAATTTGAGAATATATTCGATAAATCCGTTTTGAAAAAGTTATCTCGTAGACATTACACTGGCTGGGGTAAGTTATCTGCTAAGCTTATTAATGGTATTCGAGATGAAAAATCTGGTAATACTATTCTTGATTACTTAATTGATGATGGTATTTCTAACCGTAATTTCATGCAACTTATTCACGATGATGCTCTTTCTTTTAAAAAGAAGATACAGAAAGCACAAATTATTGGTGACGAAGATAAAGGTAATATTAAAGAGGTCGTTAAGTCTTTGCCAGGTAGTCCTGCGATTAAAAAAGGTATTTTACAAAGCATAAAAATTGTAGATGAATTGGTCAAAGTAATGGGAGGAAGAAAACCCGAGTCAATTGTTGTTGAGATGGCTCGTGAAAATCAATATACCAATCAAGGTAAGTCTAATTCCCAACAACGCTTGAAACGTTTAGAAAAATCTCTCAAAGAGTTAGGTAGTAAGATACTTAAGGAAAATATTCCTGCAAAACTTTCTAAAATAGACAATAACGCACTTCAAAATGATCGACTTTACTTATACTATCTTCAAAATGGAAAAGATATGTATACCGGAGATGATTTAGATATTGATAGATTAAGTAATTATGATATTGATCATATTATTCCTCAAGCTTTTTTGAAAGATAATTCTATTGACAATAAAGTACTTGTTTCATCTGCTAGTAACCGTGGTAAATCAGATGATGTTCCAAGTTTAGAGGTTGTCAAAAAAAGAAAGACATTTTGGTATCAATTATTGAAATCAAAATTAATTTCTCAACGAAAATTTGATAATCTGACAAAAGCTGAACGGGGAGGATTGTCACCTGAGGACAAAGCTGGTTTTATTCAACGCCAGTTGGTTGAAACACGTCAAATAACAAAACATGTAGCTCGTTTACTTGATGAGAAATTTAATAATAAAAAAGATGAAAATAATAGAGCGGTACGAACAGTAAAAATTATTACCTTGAAATCTACCTTAGTTTCTCAATTTCGTAAGGATTTTGAACTTTATAAAGTTCGTGAAATCAATGATTTTCATCATGCTCATGATGCTTACTTGAATGCCGTTGTAGCAAGTGCTTTACTTAAGAAATACCCTAAACTAGAGCCAGAATTTGTGTACGGTGATTATCCAAAATACAATAGTTTTAGAGAAAGAAAGTCCGCTACAGAAAAGGTATATTTCTATTCAAATATCATGAATATCTTTAAAAAATCTATTTCTTTAGCTGATGGTAGAGTTATTGAAAGACCACTTATTGAGGTAAATGAGGAGACCGGCGAATCCGTTTGGAATAAAGAATCTGATTTAGCAACTGTAAGGAGAGTACTCTCTTATCCGCAAGTAAATGTTGTGAAAAAAGTTGAGGAACAGAATCACGGATTGGATAGAGGAAAACCAAAGGGATTGTTTAATGCAAATCTTTCCTCAAAGCCAAAACCAAATAGTAATGAAAATTTAGTAGGTGCTAAAGAGTATCTTGACCCCAAAAAGTATGGGGGGTATGCTGGAATTTCTAATTCTTTTACTGTTCTTGTTAAAGGGACAATTGAAAAAGGTGCTAAGAAAAAAATAACAAATGTACTAGAATTTCAAGGTATTTCTATTTTAGATAGGATTAATTATAGAAAAGATAAACTTAATTTTTTACTTGAAAAAGGTTATAAAGATATTGAGTTAATTATTGAACTACCTAAATATAGTTTATTTGAACTTTCAGATGGTTCACGTCGTATGTTGGCTAGTATTTTGTCAACGAATAATAAGAGGGGAGAGATTCACAAAGGAAATCAGATTTTTCTTTCACAGAAGTTTGTGAAATTACTTTATCATGCTAAGAGAATAAGTAACACAATTAATGAGAATCATAGAAAATATGTTGAGAACCATAAAAAAGAGTTTGAAGAATTATTTTACTACATTCTTGAGTTTAATGAGAATTATGTTGGAGCTAAAAAGAATGGTAAACTCTTAAACTCTGCCTGCAAAATCATAGTATAGATGAACTCTGTAGTAGTTTTATAGGACCTACCGGAAGTGAAAGAAAGGGGCTATTTGAATTAACCTCTCGTGGAAGTGCTGCTGATTTTGAATTTTTAGGTGTTAAAATTCCAAGGTATAGAGACTATACCCCATCATCCCTATTAAAAGATGCCACACTTATTCATCAATCTGTTACAGGCCTCTATGAAACACGAATAGACCTTGCCAAACTAGGAGAGGGTTAATAAATTGGTAATAAGTATAGATAGTCTTGAGTTATTTCAAGACTATCTTTTAGTATTTAGTAGTTTCTGTATGAAGTTGAATGGGATAATCATTTTGTTAGAGAGTAGATTATAAGGATTTGATAGAGGAGGAATTAAGTTGCTTGACATATGATTATTAAGAAATAATCTAATATGGTGACAGTCACATCTTGTCTAAAACGTTGATATATAAGGATTTTTAAGGTATAATAAATATAAAAATGGAATTATTTTGAAGCTGAAGTCATGCTGAGATTAATAGTGCGATTACGAAATCTGGTAGAAAAGATATCCTACGAGGTTTTAGAGCTGTGTTGTTTCGAATGGTTCCAAAACTACGGCAGCCAACGGCTTGTTTAAGGGTCTGTTTTAGAGCTGTGTTGTTTCGAATGGTTCCAAAACATCGTCCTTCGCCGATTCAGTAAATTTGCTGTTTTAGAGCTGTGTTGTTTCGAATGGTTCCAAAACGGTCAGACAATAGGATAGAAAACTTAAGGAGTTTTAGAGCTGTGTTGTTTCGAATGGTTCCAAAACTTGCGAGTACGCAATTGACCTTTCTGTTAAGTTTTAGAGCTGTGTTGTTTCGAATGGTTCCAAAACTGGTATCTTCACTGGCCAGATTTATCTTGTGTTTTAGAGCTGTGTTGTTTCGAATGGTTCCAAAACAGTCAGGGCAAATGGGCATCAAGTTCGATGGTTTTAGAGCTGTGTTGTTTCGAATGGTTCCAAAACGCAAATTGGCGTAGAGAAAGCCGATGCCGCGTTTTAGAGCTGTGTTGTTTCGAATGGTTCCAAAACGGCCGCTCTGGTTGCTGTTGCTGCTGAAGCTTTTGTTATCACAATTTTCGGTTGACATCTCTTAGAACTCATCTTATCATAAAGGAGTCTAGTATTGAAATGTGAGAAGGGACATGTTATACGAATATCCAGCGGATCCATGC

**Step 11.** Blunt end ligation after EcoRV digestion of pACYC184 was not working, so I am switching to sticky end ligaiton. The full CRISPR+Linker cassette was re-amplified with primers containing SalI and BamHI overhangs.

CRISPR-DTL SalI F- 5’- ATGCGTCGACGTCATTGAGATCTTGGATG -3’

CRISPR-DTL BamHI R- 5’- ATGCGGATCCGCTGGATATTCGTATAAC -3’

Cut sites are underlined, priming sites are orange.

pACYC184 was then digested with SalI and BamHI, as was the custom CRISPR cassette, and sticky end ligations were performed.
